# Ethmoidal osteoid osteoma with orbital and intracranial extension – a case report

**DOI:** 10.1186/1472-6815-5-2

**Published:** 2005-03-11

**Authors:** S Balaji Pai, K Harish, MS Venkatesh, Udayshankar, Deepthi Jermely

**Affiliations:** 1 Department of Neurosurgery, M.S. Ramaiah Medical College, Gokula, Bangalore 560054, India; 2 Department of Surgical Oncology, M.S. Ramaiah Medical College, Bangalore, India; 3 Department of Plastic Surgery, M.S. Ramaiah Medical College, Bangalore, India; 4 Department of Pathology, M.S. Ramaiah Medical College, Bangalore, India

## Abstract

**Background:**

Osteoid osteoma is a benign bone neoplasm which is seen in the long bones of appendicular skeleton. It is rarely seen in the cranium. Skull base osteoid osteoma is extremely rare and has been anecdotally reported.

**Case presentation:**

The authors report a case of a large osteoid osteoma of the ethmoid with intraorbital and intracranial extension in a 33 year old male patient. He presented with loss of vision in the left eye. The intra-cranial extension was excised through a single burr-hole fronto-orbitotomy. The ethmoid and orbital portions were approached and excised through a Weber-Ferguson incision and inferior orbitotomy. Radical excision of the tumor could thus be achieved through a craniofacial approach.

**Conclusion:**

Although benign and rare, skull base osteoid osteoma can present with neurological deficit due to its mass effect and involvement of vital structures. A multispeciality team approach is advisable in such cases if radical excision is to be achieved. A craniofacial approach made radical single stage excision of this multicompartmental osteoid osteoma possible with an uneventful postoperative period.

## Background

Osteoid osteoma is a rare benign osteoblastic lesion usually involving the long bones of the lower limbs. Cranial involvement has been mainly localised to the skull vault. Osteoid osteoma of the skull base is a rare entity [1]. Surgical management of skull base osteoid osteoma may be challenging due to its proximity to vital structures, access and hard consistency. This report deals with a case of an ethmoidal osteoid osteoma invading the adjacent orbit and anterior cranial fossa and the team approach employed to achieve radical excision. The radiological findings and the surgical procedure employed are presented.

## Case presentation

### Clinical presentation

**Figure 1:**
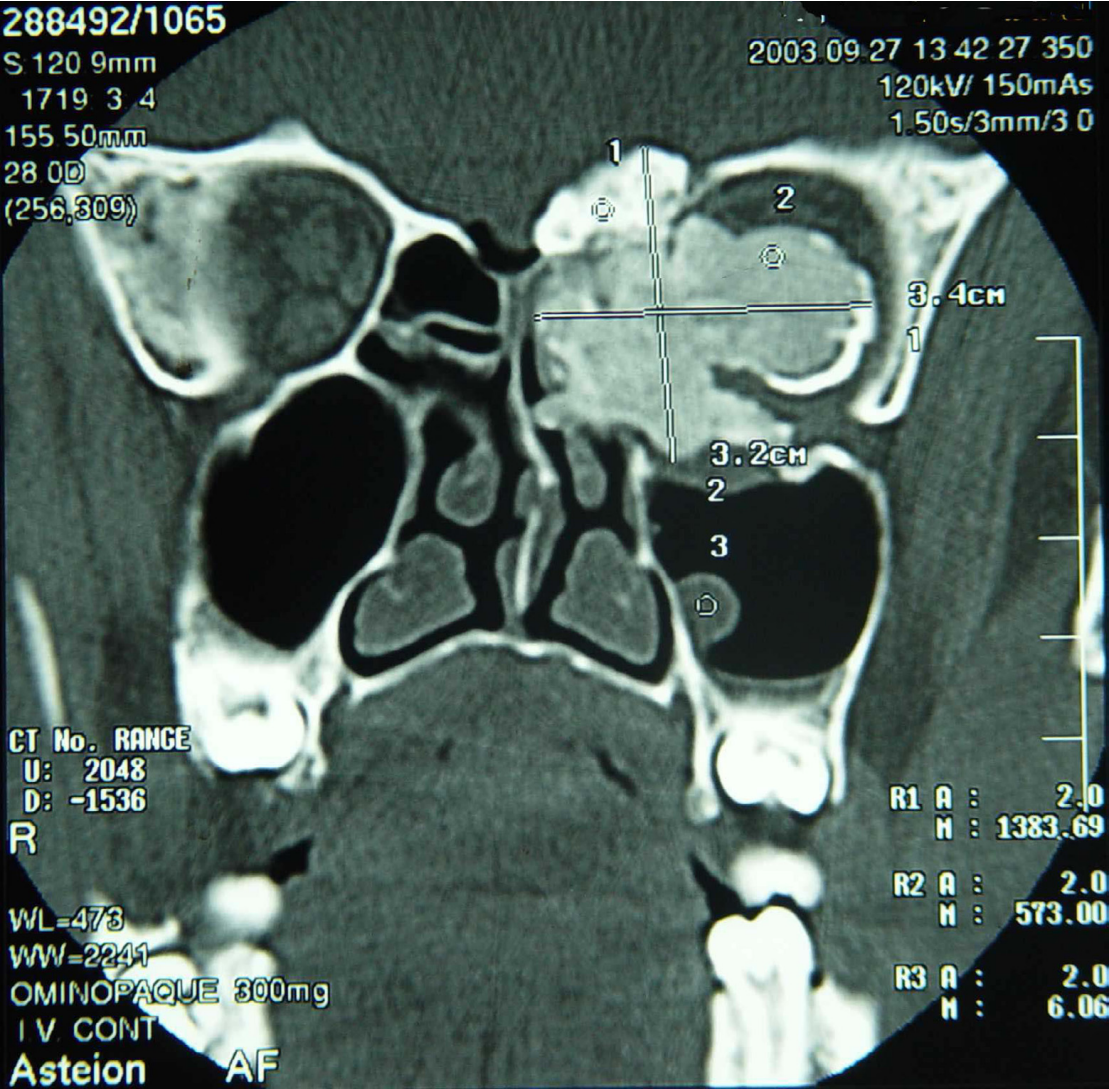
**Preoperative CT scan. **Preoperative coronal CT scan which shows a large bony tumor arising from the left ethmoid sinus with orbital and intracranial extension.

A 33 year old male presented with decreased vision in the left eye and left sided headache of 3 months duration. Examination revealed left eye blindness with primary optic atrophy and no other neurological deficit. Imaging revealed a bony tumor in the left ethmoid sinus invading the left orbit and compressing the left optic nerve. Intracranial extension into the anterior cranial fossa on the left side was noted (Figure 1). Core biopsy of the mass showed an osteoid osteoma.

### Surgical management

**Figure 2:**
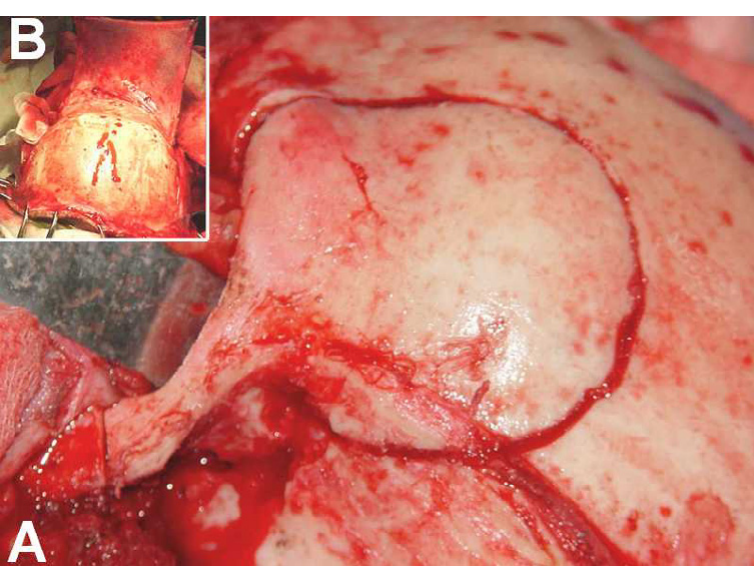
**Elevation of pericranial flap and craniotomy. **Pericranial flap being raised after elevation of the scalp flap (B) followed by a single burr hole frontoorbitotomy (A).

**Figure 3:**
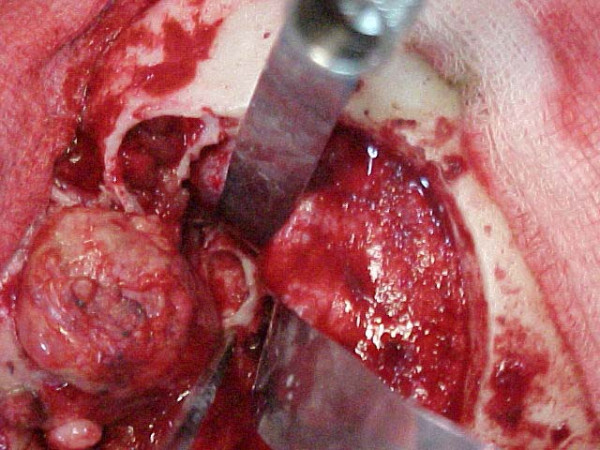
**Intracranial portion of the tumor. **Dura being retracted after the craniotomy and visualization of the intracranial portion of the tumor. The tumor core has been drilled. The shell was subsequently nibbled away.

**Figure 4:**
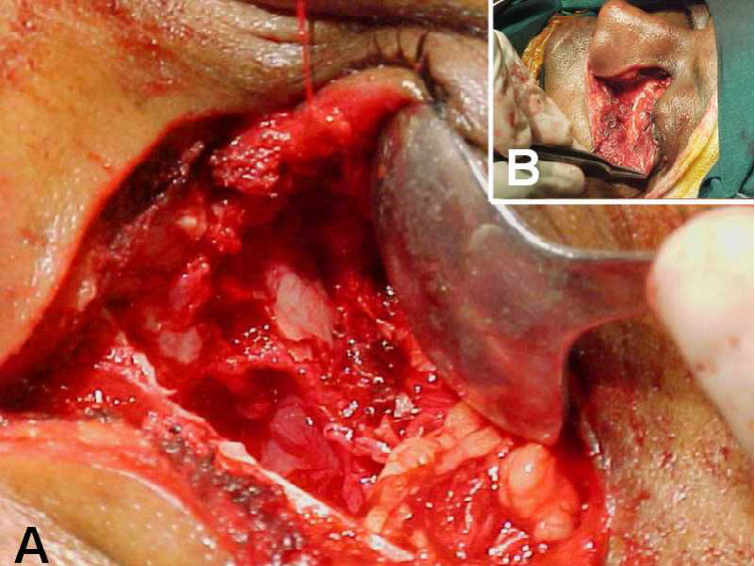
**Facial approach **Weber Ferguson incision (B) followed by inferior orbitotomy (A) which enabled the retraction of the eyeball.

**Figure 5:**
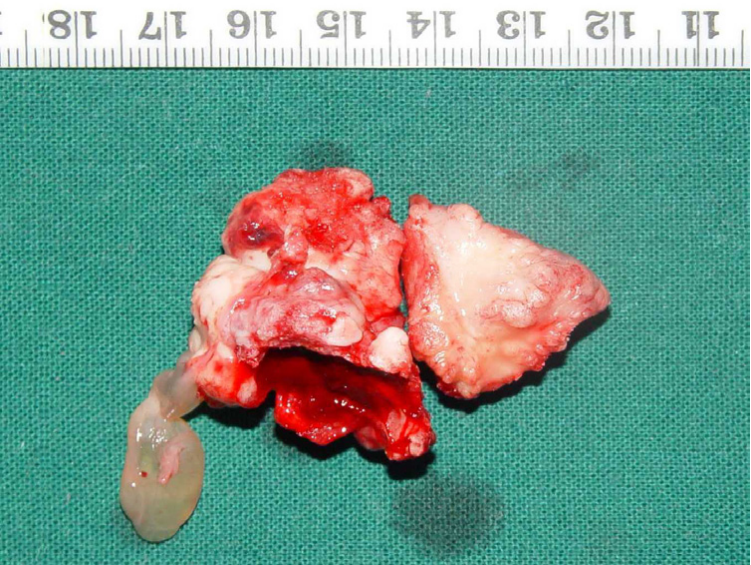
**Specimen. **Specimen after it was excised in 2 pieces with part of the ethmoid sinus mucous membrane attached to one of the pieces. The ethmoidal portion was subsequently drilled away.

A multispeciality team approach was devised to achieve radical excision of the tumor. Bicoronal scalp flap and pericranial flaps were raised separately. A single burr hole left fronto orbital bone flap was raised including the orbital roof and left zygomaticofrontal process (Figure 2). Dura was retracted and the bony hard whitish tumor visualised. This was excised using the high speed drill (Figure 3). Weber Ferguson incision was used to access the orbital portion of the tumor. Medially the tumor could be felt in the orbit but retraction of the globe was difficult. Hence inferior orbitotomy was done by removing the lower and lateral orbital margins. The intraorbital contents could now be retracted laterally and the tumor visualized (Figure 4). The tumor was then detached from the ethmoid sinus and the intraorbital extension excised. The ethmoidal portion was drilled and radical excision achieved (Figure 5). Dural tears were covered with temporalis fascia and glue. The ethmoidal sinus was packed with free temporalis muscle graft. Vascularised pericranial graft was used to cover the anterior skull base. The frontal sinus was exenterated and packed with gelfoam. The bone flap and orbital margins were replaced.

### Postoperative period and follow up

**Figure 6:**
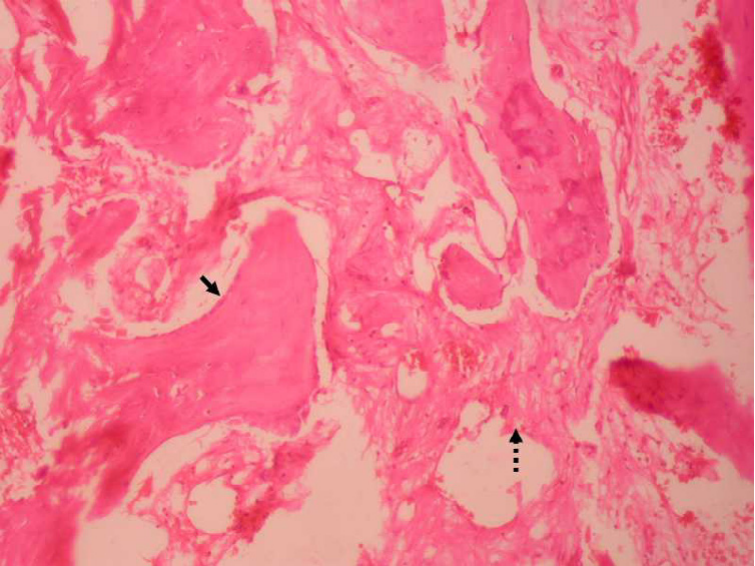
**Microphotograph of the tumor. **Microphotograph (250X) of the specimen in H & E stain showing new osteoid lined by plump osteoblasts (solid arrow) in a highly vascularised stroma (broken arrow).

**Figure 7:**
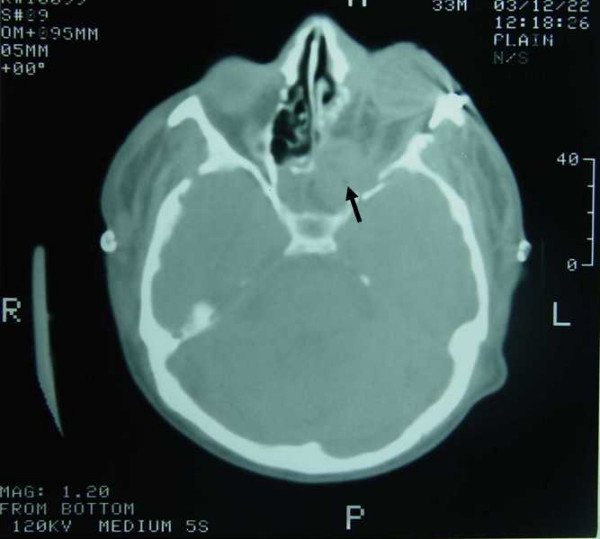
**Postoperative CT. **Postoperative CT scan of the patient showing total excision of the tumor and the presence of free temporalis graft in the ethmoid and sphenoid sinus (arrow).

Post-operatively patient had a frozen left eye (possibly due to retraction of the orbital contents) with no improvement in the left eye vision in the immediate postoperative period. His postoperative period was otherwise uneventful. Histopathological examination revealed an osteoid osteoma (Figure 6). On follow up after 12 months patient was disease free (Figure 7).

## Discussion

Osteoid osteoma is a benign osteoblastic lesion and constitutes 1% of all bone tumors and 11% of benign bone lesions [1]. It is usually seen in the second and third decades and a male preponderance has been noted. It can occur throughout the skeleton but the long bones of the lower extremities and the vertebrae are most commonly affected. They are usually metaphyseal but may be epiphyseal occasionally. It is frequently localized to the cortex (85%) but may also occur in spongiosa (13%) and subperiosteal region (2%) [2]. Cranial cases are seen to generally arise from the skull vault. Skull base osteoid osteomas are extremely rare and occur in the frontal or ethmoidal sinuses [1,3]. It usually presents with sharply localized pain and tenderness especially at night. In our case the osteoid osteoma was seen to originate from the ethmoid sinus and pain was not a presenting feature.

The radiological diagnosis rests on Computerised Tomography and isotope bone scan [1]. Radiographically osteoid osteoma appears as a radio opaque lesion with a nidus which has a radiolucent centre surrounded by dense sclerosis [2]. This may at times be mistaken for Garre's osteomyelitis. Occasionally the nidus may have a radio opaque centre with a surrounding radiolucent area. In our patient no definite nidus could be visualized probably due to the large size and unusual location.

The treatment generally consists of en bloc resection or curettage of the tumor. Recurrence rate after incomplete resection may be upto 10% [1]. If asymptomatic and small, the lesion may be left alone and observed. However a rare complication of a large pneumocephalus has been reported by Ferlito et al from a frontoethmoidal osteoid osteoma [4]. Our patient presented with loss of vision due to a large osteoid osteoma of the ethmoid invading the left orbit. Anterior skull base lesions have been approached through a frontoorbitotomy which is usually removed as two separate parts. We have found that a single burr hole frontoorbitotomy flap gives excellent exposure to the anterior skull base without excessive retraction on the brain and is also cosmetically superior. Combining the craniotomy with a Weber Ferguson incision and orbitotomy made a single stage radical excision possible.

Histologically the nidus is sharply delineated from the surrounding variably thick layer of dense bone. The nidus is composed of more or less calcified osteoid lined by plump osteoblasts within a highly vascularised connective tissue stroma [2] (Figure 6). It does not invade the adjacent tissue. No malignant transformation has been reported [1]. A differential diagnosis of benign osteoblastoma may be entertained. However, in the case of the latter, active osteoblasts are more numerous and the stroma is richly vascularized and extravasated blood with large number of multinucleated giant cell macrophages are noted [5]. Several authors have stressed the fact that the two are identical histologically and the differentiation between them if any can only be on the basis of size [5]. In our patient although the tumor was large, none of the above mentioned histological features of a benign osteoblastoma could be noted.

## Conclusion

Osteoid osteoma of the skull base is rare and anecdotally reported. Radical excision is difficult especially if the tumor involves major blood vessels and cranial nerves. The surgical team constituted the neurosurgeon, surgical oncologist and plastic surgeon. A craniofacial approach made radical single stage excision of this multicompartmental osteoid osteoma possible with an uneventful postoperative period.

## Competing interests

All the authors of the article "ETHMOIDAL OSTEOID OSTEOMA WITH ORBITAL AND INTRACRANIAL EXTENSION -CASE REPORT" hereby declare that there are no competing interests – financial and non financial.

## Authors' contributions

**SBP **performed the craniotomy, excised the intracranial extension of the tumor, assisted in excising the orbital portion, drilled the ethmoidal portion and drafted the manuscript.

**KH **excised the orbital portion and helped draft the manuscript.

**MSV and US **performed the orbitotomy, helped in tumor excision and closure.

**DJ **did the histopathological examination and helped in drafting the manuscript.

*All authors have read and approved of the manuscript*.

## Pre-publication history

The pre-publication history for this paper can be accessed here:
